# Investigation of pyrimidine nucleoside analogues as chemical probes to assess compound effects on the proliferation of *Trypanosoma cruzi* intracellular parasites

**DOI:** 10.1371/journal.pntd.0008068

**Published:** 2020-03-12

**Authors:** Melissa Louise Sykes, David Hugh Hilko, Livia Isabella Kung, Sally-Ann Poulsen, Vicky Marie Avery

**Affiliations:** 1 Discovery Biology, Griffith Institute for Drug Discovery, Griffith University, Nathan, Australia; 2 Chemical Biology, Griffith Institute for Drug Discovery, Griffith University, Nathan, Australia; 3 Institute of Molecular Health Sciences, ETH Zurich, Switzerland; Center for Biologics Evaluation and Research, Food and Drug Administration, UNITED STATES

## Abstract

*Trypanosoma cruzi* parasites utilise *de novo* pyrimidine biosynthesis to produce DNA and survive within mammalian host cells. This pathway can be hijacked to assess the replication of intracellular parasites with the exogenous addition of a DNA specific probe. To identify suitable probe compounds for this application, a collection of pyrimidine nucleoside analogues was assessed for incorporation into *T*. *cruzi* intracellular amastigote DNA using image-based technology and script-based analysis. Associated mammalian cell toxicity of these compounds was also determined against both the parasite host cells (3T3 cells) and HEK293 cells. Incorporation of 5-ethynyl-2′-deoxyuridine (EdU) into parasite DNA was the most effective of the probes tested, with minimal growth inhibition observed following either two or four hours EdU exposure. EdU was subsequently utilised as a DNA probe, followed by visualisation with click chemistry to a fluorescent azide, to assess the impact of drugs and compounds with previously demonstrated activity against *T*. *cruzi* parasites, on parasite replication. The inhibitory profiles of these molecules highlight the benefit of this approach for identifying surviving parasites post-treatment *in vitro* and classifying compounds as either fast or slow-acting. F-ara-EdU resulted in <50% activity observed against *T*. *cruzi* amastigotes following 48 hours incubation, at 73 μM. Collectively, this supports the further development of pyrimidine nucleosides as chemical probes to investigate replication of the parasite *T*. *cruzi*.

## Author summary

Chagas disease occurs within 21 countries in the Americas, causes over 10, 000 deaths per year and a further 25 million people are at risk of being infected. The cause of Chagas disease is *Trypanosoma cruzi*, a single celled protozoan parasite, which enters the bloodstream of a host by the bite of a “kissing bug”. In advanced disease stages, the parasite hides in heart and gut tissue and is difficult to treat. Identifying the replicative ability of these parasites is important to understanding Chagas disease progression and the effectiveness of compounds and drugs for treatment. By testing a panel of nucleoside analogues that may incorporate into DNA during synthesis, we developed an image-based method with a fluorescently-labelled DNA probe to identify replicating parasites. This method has effectively shown that drugs used to treat the parasite are able to clear intracellular infection, whilst a compound that was not efficacious in clinical trials leaves replicating *T*. *cruzi* behind. This methodology can be used to understand the action of further compounds and supports the identification of new, less toxic probes to assess intracellular parasite replication.

## Introduction

Chagas disease, caused by the protozoan parasite *Trypanosoma cruzi*, is considered one of the world’s 20 most neglected tropical diseases [1]. The disease is endemic to 21 countries within the Americas, and causes more than 10 000 deaths per year, with a further 25 million people at risk of acquiring the disease [2]. The nitro-heterocyclic drugs that are currently used to treat *T*. *cruzi* infection, nifurtimox (NFX) and benznidazole (BZ), have questionable efficacy in the chronic stage and associated toxicity often leads to cessation of treatment [3]. New drugs are therefore needed, however high attrition rates in the drug discovery pipeline remains an issue [4]. Lack of efficacy of the azole antifungal cytochrome P450 (CYP51) inhibitors, posaconazole and ravuconazole (E1224), in the treatment of chronic Chagas infection [5,6] has highlighted the need to understand more about the action of compounds on parasite replication. Improving efficacy and knowledge of the mode of action of new compounds effective against the parasite would support and accelerate the discovery of new drugs against Chagas disease.

We have previously developed a sensitive high-throughput, high-content assay to assess compound activity against intracellular *T*. *cruzi* parasites. This image-based technique can detect as few as 5 parasites per host cell with the relative clearance of parasite populations [7] determined utilising Hoechst, a sensitive marker for *T*. *cruzi* and host cell genomic DNA structure, in combination with HCS CellMask Green. Metabolic assays, which can be used to determine the static / cidal MOA of compounds against axenic, extracellular parasites [8] are incompatible with identifying metabolising / replicating *T*. *cruzi* intracellular amastigotes. We and others have identified *T*. *cruzi* parasites remaining in host cells following treatment with CYP51 inhibitors for 48–96 hours [9–11]. It would be of tremendous benefit to drug discovery campaigns if replicating *T*. *cruzi* parasites could be distinguished from non-replicating parasites following compound treatment. Determining the replicative ability of remaining parasites following compound exposure can distinguish cells that are still viable. This would aid in identification of compounds that may inhibit cell division but do not kill (potentially static mode of action), or populations of cells that are resistant to treatment. Either case could cause lack of treatment efficacy and thus are important to identify when prioritising compounds. This can in principle be undertaken by analysis of DNA replication in the parasite by incorporation and detection of nucleoside analogues during DNA biosynthesis, a method which has been utilised for a number of eukaryotic cells [12].

The replication of *T*. *cruzi* extracellular epimastigote forms, in relation to their cell cycle, has previously been assessed using immunofluorescence with the thymidine analogue, 5-bromodeoxyuridine (BrdU), in combination with an anti-tubulin monoclonal antibody and the nuclear marker, DAPI, to determine the timing of kinetoplast and flagellar pocket division [13]. Additionally, BrdU has been utilised to determine the level of DNA carried from *T*. *cruzi* trypomastigotes to amastigotes during amastigogenesis [14], and indirectly to investigate the effect of *T*. *cruzi* infection upon the mammalian cell cycle [15]. BrdU incorporates into the S-phase of dividing eukaryotic cells during DNA synthesis and has been applied in mammalian cell studies to determine cell fate, cell origin, cell migration and cell cycle events [16]. Detection of BrdU labelling requires denaturation of DNA into single strands to allow the BrdU-specific antibody access to its binding site. The detection procedure is time-consuming, while the harsh conditions can degrade specimen structures, distort the cell and tissue structure and affect the intensity of staining [17].

An alternative method for studying replication of eukaryotic cells is by integration of the nucleoside analogue, 5-ethynyl-2′-deoxyuridine (EdU), into DNA during *de novo* DNA synthesis. EdU is incorporated into DNA during the S-phase of the cell cycle. The terminal alkyne group of EdU is exposed and accessible to reagents enabling the level of DNA incorporation to be quantified by reaction with a fluorescent small molecule azide using Cu^I^ catalysed azide–alkyne cycloaddition (CuAAC, or click chemistry) [17,18]. Because of its small size and physicochemical properties compatible with membrane permeability, the fluorescent azide diffuses through live and fixed tissues and readily accesses genomic DNA. Incorporation of EdU into parasite DNA, detected with click chemistry, has recently been used as a chemical probe to identify replicating *T*. *cruzi* parasites, to assess variation in strain replication and responses to a selection of compounds at a single concentration [19]. Additionally, an image-based assay to assess parasite replication utilising EdU as a chemical probe was developed to determine compound activity against *Leishmania donovani* [20]. The incorporation of EdU and BrdU has been compared in *T*. *cruzi* epimastigotes and it was found that EdU was most effective for monitoring DNA replication because of its sensitivity and that DNA denaturation is not required [21].

Studies have shown that both BrdU and EdU can be toxic *in vitro* to some breast cancer cell lines following long-term *in vitro* treatment (96 hours), with these effects being cell type specific and concentration dependant [22]. Generally, toxicity was ameliorated with shorter pulse incubations, however there is the need to independently evaluate and optimise incubation time and nucleoside analogue concentration before applying probes to assess cellular proliferation in new cell types. In this study, we evaluated the activity of a set of ethynyl pyrimidine nucleoside analogues against intracellular *T*. *cruzi* parasites. We then assessed DNA incorporation using the safe concentration determined of the probe [23], followed by image-based analysis. The effect of a small collection of compounds and approved drugs with known activity against *T*. *cruzi* intracellular amastigotes on parasite replication [7] was then determined using this new assay. Test compounds and drugs representative of differing modes of action (MOA) were selected for evaluation, including standard-of-care drugs nifurtimox and benznidazole, the recent clinical candidate posaconazole (a CYP51 inhibitor against *T*. *cruzi* [24]). Additionally, clemastine fumarate, with an unknown MOA against the parasite [9], was profiled for effects upon *T*. *cruzi* replication. Our results show that ethynyl pyrimidine nucleoside analogues are indeed promising chemical probes for visualising inhibitor effects on intracellular *T*. *cruzi* parasite replication. Nucleoside analogue incorporation into parasite DNA enables the determination of compound efficacy and speed of action against *T*. *cruzi* replication *in vitro*. The profile of known drugs and compounds effective against *T*. *cruzi* replication reveals that different classes of inhibitors can be classified as either slow-or fast-acting inhibitors of parasite replication.

## Methods

### Growth and maintenance of parasites and cell lines

Parasites and host cells were maintained as outlined previously [7]. Briefly, host 3T3 fibroblasts (ATCC, CCL-92) were grown in RPMI medium (Thermo Fisher Scientific, USA) with no phenol red, supplemented with 10% FBS (Thermo Fisher Scientific, USA) and incubated at 37°C in 5% CO_2_. Host cells were harvested at 70% confluency and were split every 2–3 days in 175 cm^2^ flasks. 3T3 cells were cultured up to passage 7 as contact inhibition was reduced or lost at higher passages. Prior to infection with *T*. *cruzi* trypomastigotes, host cells were seeded at 1.2 x 10^6^ cells in 75 cm^2^ or 4 x 10^5^ cells in 25 cm^2^ flasks and grown for 24 hours before parasite addition. Parasites were added at a multiplicity of infection (MOI) of 10:1 and incubated for 24 hours before non-infected trypomastigotes were washed off the host cell bed with PBS supplemented with Ca^2+^ and Mg^2+^ (Thermo Fisher Scientific, USA). Incubation was continued for a further 3 days, until tissue culture trypomastigotes began to egress from host cells. Egressed parasites were used for assays or to continue the infective life cycle *in vitro*.

For determination of compound activity against a replicating mammalian cell line, human embryonic kidney cells (HEK293, ATCC) were grown in high glucose DMEM medium (Thermo Fisher Scientific, USA) supplemented with 10% FBS at 37°C and 5% CO_2_. Cells were sub-cultured every 3 or 4 days by seeding 1.5 x 10^6^ or 1 x 10^6^ cells respectively, into 175 cm^2^ flasks.

### Compounds

**Fig 1:**
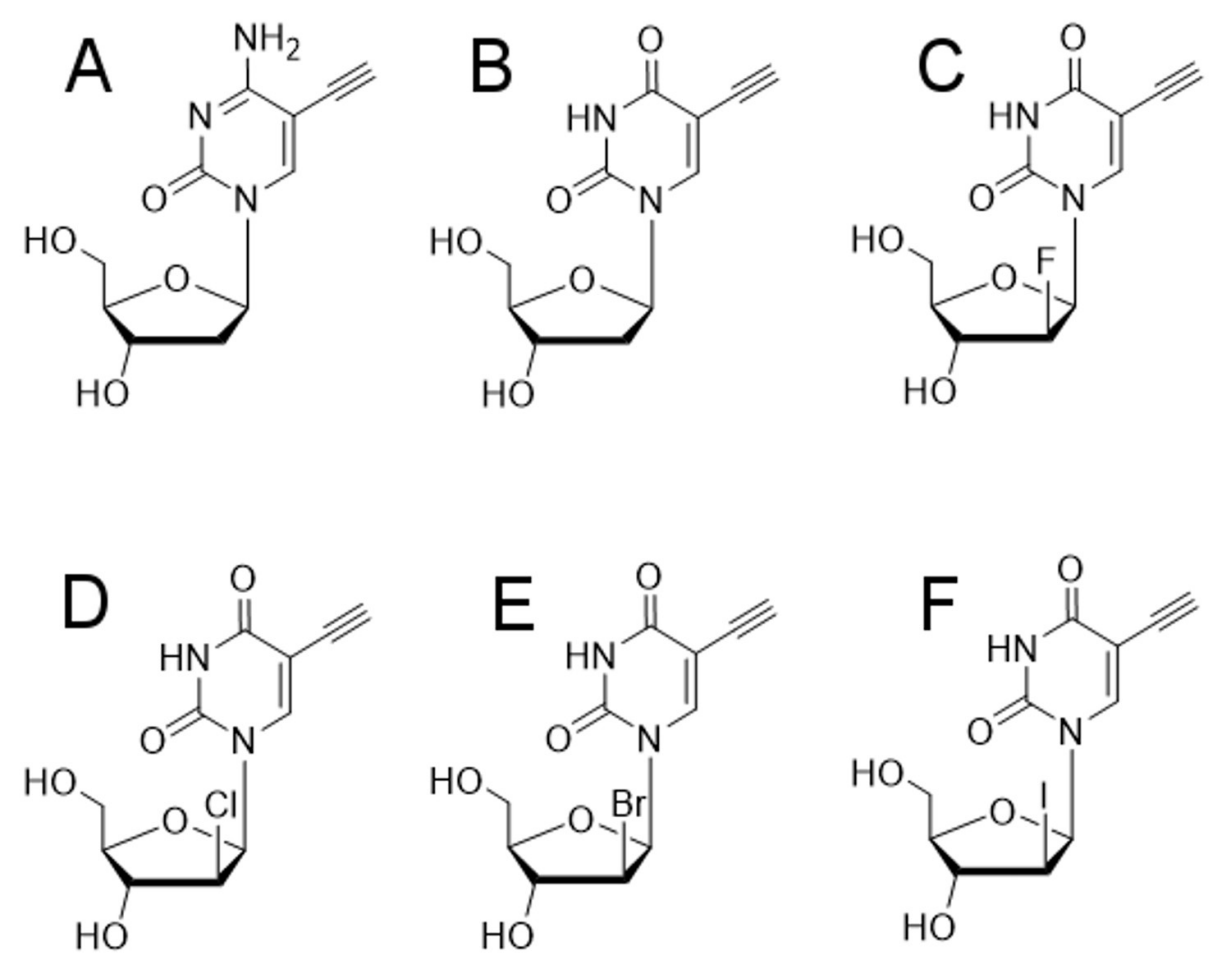
Chemical structures of the nucleoside analogues in this study. A) EdC. B) EdU. C) F-ara-EdU. D) Cl-ara-EdU. E) Br-ara-EdU. (F) I-ara-EdU.

Chemical structures of EdU (5-ethynyl-2′-deoxy-uridine); EdC (5-ethynyl-2′-deoxy-cytidine); F-ara-EdU ((2*S*′)-2′-deoxy-2′-fluoro-5-ethynyl-uridine); Cl-ara-EdU ((2′*S*)-chloro-2′-deoxy-5-ethynyl-uridine); Br-ara-EdU ((2′*S*)-2′-bromo-2′-deoxy-5-ethynyl-uridine); and I-ara-EdU (2′*S*)-2′-deoxy-5-ethynyl-2′-iodo-uridine) are shown in Fig 1. EdU, Cl-ara-EdU, Br-ara-EdU, and I-ara-EdU were synthesised as described previously [23,25]. F-ara-EdU and EdC were purchased from Sigma Aldrich (USA). Compound stocks were prepared at 20 mM concentration for incorporation studies, or 50 mM for temporal studies, in 100% DMSO.

Test compounds used to determine the effect on parasite replication were purchased from the suppliers as indicated clemastine fumarate (CF, Sapphire Biosciences, USA), posaconazole (POSA, Sigma Aldrich, USA), nifurtimox (NFX), and benznidazole (BZ, kindly provided by Epichem Australia). NFX was extracted from Lampit tablets by Dr Agatha Garavalas whilst at the Griffith Institute for Drug Discovery (Lampit kindly supplied by the Drugs for Neglected Diseases initiative; DND*i*). Compounds were prepared at stock concentrations of 7.0 and 0.27 mM in 100% DMSO for CF and POSA, respectively, whilst NFX and BZ were prepared at 35 mM.

### Image-based assay to assess the effect of nucleoside analogues on the number of *T*. *cruzi* infected cells over time and following compound removal

The activity of the nucleoside analogues against *T*. *cruzi* intracellular amastigotes following 24, 48 hours exposure and compound wash off, was determined using image-based techniques as previously described [9]. Briefly, 14-point serial dilutions of the compounds in 100% DMSO were prepared and then further diluted in sterile water at a ratio of 1:21 compound: water. Five microliters of the diluted compounds were added to wells of *T*. *cruzi* infected host cells, using a MiniTrak compound handling device (Perkin Elmer, USA) with final concentrations in the plate ranging from 183 μM to 4.6 x 10^−4^ μM. All experiments were performed with two separate biological replicates.

### Redox-based assay to assess HEK293 viability

To determine the effect of the nucleoside analogues against a replicating mammalian cell line, analogues were tested against HEK293 cells, utilising a REDOX-based metabolic assay to assess cell viability as previously described [26]. IC_50_ values were calculated from two separate biological experiments, utilising 30 μM puromycin (PURO) as a positive control and 0.40% DMSO as a negative control. Experiments were performed over two biological replicates.

### Pyrimidine nucleoside analogues as DNA probes to identify *T*. *cruzi* replication

*T*. *cruzi* infected 3T3 fibroblasts were initially exposed to nucleoside analogues for 48 hours at concentrations ranging from 73 μM to 1.8 x10^-4^ μM. Host cell, parasite and compound additions were carried out as previously described [7]. Cells were then stained using a click chemistry azide cocktail, followed by Hoechst to identify nuclear material, as previously reported [23]. The freshly prepared azide cocktail consisted of a final concentration of 5 μM of Alexa Fluor 488 azide (Thermo Fisher Scientific, USA), 1 mM copper sulfate (Sigma Aldrich, USA) and 100 mM sodium ascorbate (Sigma Aldrich, USA), in DPBS. Wells were imaged on an Opera image-based reader (Perkin Elmer, USA) at 20x magnification with 405 nm (defined as Hoechst channel) and 488 nm (defined as Alexa 488 channel) lasers using filter sets/bandwidth (emission) of 450/50 nm and 540/75 nm, respectively. To determine nucleoside analogue incorporation into *T*. *cruzi* amastigote DNA, a script was developed utilising the Columbus (PerkinElmer, USA) image-based analysis and data handling platform (CHEMICAL LABEL INCORPORATION SCRIPT). Nucleoside analogues were also tested for DNA incorporation following 2, 4 and 24 hours exposure, at the same concentrations. All experiments were performed in duplicate. The colocalisation of nucleoside analogues and Hoechst (replicating parasites) at each time point (48, 4 and 2 hours) was compared using GraphPad Prism 5.

### Detection of *T*. *cruzi* replication following exposure to inhibitors

The effect of compounds with known activity against *T*. *cruzi* (CF, POSA, NFX and BZ) on *T*. *cruzi* replication was assessed by measurement of EdU incorporation into parasite DNA following 48 and 2 hours exposure to compounds. The addition of host cells, parasites and compounds were as described for the *T*. *cruzi* image based method previously reported [7]. Final assay concentrations ranged from 26 to 6.4 x 10^−4^ for CF, POSA 1.0 ± 5.0 x 10^−5^ μM, NFX and BZ from 127 to 3.2 x 10^−4^ μM. Following compound wash off and addition of 50 μL of RPMI medium to each well on a Bravo liquid handler (Agilent Technologies, USA), 5 μL of EdU diluted 1:21 in sterile H_2_O, at a final assay concentration of 0.37 μM was added to wells and incubated for 4 hours (following 48 hours exposure to compounds) or 1.83 μM (following 2 hours exposure to compounds). Plates were incubated for either 4 or 2 hours at 37°C in 5% CO_2_ before click chemistry cocktail and Hoechst fluorescent staining [23], with the addition of 0.005x Cell Mask Deep Red Plasma Membrane stain to identify the cytoplasm of the host cells. Addition of nuclear and cytoplasmic dyes was undertaken as previously reported [7] and images captured on an Opera confocal image-based system at 20x magnification.

Image analysis was performed with a script developed in Columbus software (CHEMICAL LABEL INCORPORATION SCRIPT). Control wells were also imaged at an increased, 60x magnification to show the distribution of staining and the structure of the parasites within host cells. Eight background control wells consisted of infected host cells, fixed and exposed to Hoechst and the click chemistry cocktail, without the addition of the chemical probe EdU. IC_50_ values of the inhibitors with known activity were calculated by exporting the number of spots that showed staining with Hoechst in infected cells, and separately the number of Hoechst identified spots incorporating Alexa Fluor 488, with fluorescence intensity above background (in infected cells). The percentage activity of compounds against parasite number / infected cells (identified with Hoechst) was calculated against a positive control of 12 μM of NFX for the parasite and 30 μM of PURO the host cells; and a negative control of 0.47% DMSO. All images taken were not modified and were taken on an Opera, confocal image-based reader.

### IC_50_ analysis, calculation of % activity at E_max_ and statistical analysis

IC_50_ values for inhibitory activity of both the nucleoside analogues tested as DNA probes and known inhibitors tested to determine their effect on replication of *T*. *cruzi* were calculated in GraphPad Prism 5, using sigmoidal dose response with variable slope. Results are given ± standard deviation and mean values were taken from two experimental replicates. The p-value for difference in the % activity at E_max_ concentrations for compounds with known activity (EdU identified parasites, compared to Hoechst identified parasites) against *T*. *cruzi* was determined using GraphPad Prism 5, using an unpaired student’s t-test. P-values of <0.05 were considered significantly different. The E_max_ for each compound was at least two data points in from the beginning of the plateau of activity in the IC_50_ curve, as previously described [9].

## Results

### Activity of pyrimidine nucleoside analogues against *T*. *cruzi*, 3T3 and HEK293 cells

Prior to determining incorporation competency of the six pyrimidine nucleoside analogues (Fig 1) into the DNA of *T*. *cruzi* amastigotes, the effect of these compounds on the growth of intracellular parasites and 3T3 host cells over time was investigated. EdU and EdC exhibited activity against *T*. *cruzi* following 24 hours incubation, with the IC_50_ value decreasing following 48 hours incubation, with a 1.8 fold increase in activity for both EdC and EdU. The IC_50_ values of EdU and EdC against *T*. *cruzi* following 48 hours incubation were 0.040 ± 0.025 μM and 7.1 ± 3.7 μM, respectively, whilst F-ara-EdU, Cl-ara-EdU, Br-ara-EdU, and I-ara-EdU had ≤50% inhibitory activity at the highest test concentration of 183 μM (Table 1). Following 48 hours incubation and compound wash off, with a further 72 hours incubation in the absence of compound, an IC_50_ value could not be determined for EdC, and there was a 10 fold increase in the IC_50_ value for EdU. The relative E_max_ was shown for each time point, both EdC and EdU displayed incomplete clearance of infected cells.

**Table 1:** The inhibition and selectivity indices (SI) of pyrimidine nucleoside analogues against intracellular *T*. *cruzi* following 48 hours incubation. SI is in relation to HEK293 cells. 1) IC_50_ value, or if IC_50_ value could not be determined, the percentage inhibition at the highest test concentration of 183 μM (or for HEK293 cells this was 87 μM). NA = ≤50% activity at the highest concentration tested and an IC_50_ value could not be determined. EdU and EdC showed <20% activity against 3T3 cells in the image-based assay. 2) EdU resulted in a plateau of activity (E_max_) of 57% against HEK293 cells, therefore the IC_50_ value was estimated and based on a sub-optimal effect against these cells. 3) F-ara-EdU inhibited 50% of parasite growth at 183 μM, with <50% inhibition observed at 73 μM. Selectivity index (SI) to *T*. *cruzi* intracellular amastigotes was in relation to HEK293 cells. * As residual host cells were not completely inhibited, and although they were smaller and fibrous, they could not be excluded from the script, thus the IC_50_ value against host cells following wash off could not be determined. NFX = nifurtimox. PURO = puromycin.

Nucleoside analogue	*T*. *cruzi* 24 (μM) ^1^	*T*. *cruzi* 48 (μM) ^1^	*T*. *cruzi* Wash off (μM) ^1^	Host cell 3T3 IC_50_ value 48 h (μM)^1^	HEK293 IC_50_ value (μM) ^1^	SI
EdC	12.5 ± 2.0 (69%)	7.1 ± 3.7 (87%)	90% at 183 μM	NA	NA	> 26
EdU	0.073 ± 0.049 (83%)	0.040 ± 0.025 (98%)	0.42 ± 0.061 (99%)	NA	0.67 ± 0.26^2^	17
F-ara-EdU	NA	NA^3^	NA	NA	NA	-
Cl-ara-EdU	NA	NA	NA	NA	NA	-
Br-ara-EdU	NA	NA	NA	NA	NA	-
I-ara-EdU	NA	NA	NA	NA	NA	-
NFX	1.72 ± 0.86 (100%)	0.97 ± 0.026 (100%)	1.41 ± 1.08 (100%)	NA	NA	> 143
PURO	7.6 ± 1.2 (92%)	2.36 ± 0.78 (100%)	*	4.4 ± 0.80	0.41 ± 0.17	0.11

To determine growth effects on replicating mammalian cells, HEK293 cells were exposed to nucleoside analogues for 48 hours. EdU showed a sub-efficacious effect, with an E_max_ of 57% and thus the IC_50_ value was only estimated, and relative to this plateau of activity. Both EdU and EdC displayed selective inhibitory activity toward *T*. *cruzi*, with selectivity indices (SI) of 17 and >26, respectively following 48 hours exposure. The positive control NFX exhibited an IC_50_ value of 0.97 ± 0.026 μM. The assay reproducibility was demonstrated with a Z’-factor of 0.69 for the *T*. *cruzi* assay and 0.66 for the host 3T3 cell assay. The Z’-value for the HEK293 assay was 0.82.

The inhibitory effects of EdU and EdC against *T*. *cruzi* amastigote growth were tested following 2 and 4 hour exposures. Neither EdU nor EdC showed appreciable activity against the parasite, nor the host cells, with activity less than 20% at 73 μM. The positive assay control, NFX, (exposure for 48 hours) showed an IC_50_ value of 1.002 ± 0.101 μM. A Z’-factor of 0.73 for parasites and 0.71 for host cells indicated assay reproducibility.

### Incorporation of pyrimidine nucleoside analogues into *T*. *cruzi* DNA

To determine if nucleoside analogues incorporated into *T*. *cruzi* DNA, parasites were initially exposed to each analogue for 48 hours. Following exposure of parasites to EdU, all concentrations assessed that also incorporated the probe had a negative effect on parasite survival (activity depicted in Table 1). However, the successful labelling was used to initially generate a script to identify nucleoside analogue incorporation for further use to assess shorter incubations. Exposure of parasites to 0.037 μM of EdU was used to develop the DNA incorporation script. This concentration was selected as the lowest concentration whereby EdU could be detected, in an effort to diminish the growth suppression of *T*. *cruzi* displayed by this nucleoside analogue.

**Fig 2:**
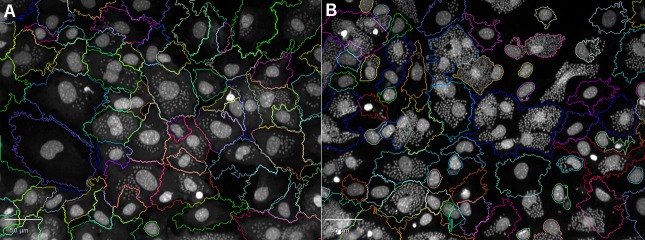
Images of 3T3 host cells illustrating the cytoplasmic borders defined after applying the chemical label incorporation script to assess colocalisation of Hoechst and EdU. Host cell cytoplasm is clearly identified by the coloured borders, and both host cell and parasite nuclei defined by Hoechst. Images were taken at 20x magnification in the Hoechst channel. A) Treatment with 0.037 μM of EdU. B) no addition of EdU.

A CHEMICAL LABEL INCORPORATION SCRIPT developed in Columbus firstly identified Hoechst stained parasites within host cells by spot analysis, utilizing the same building block analysis as we had previously developed [7] to determine *T*. *cruzi* infected cells. The physical definition of amastigote DNA was identified by Hoechst, with the only modification being that as there was no cytoplasmic marker, thus segmentation borders in the Hoechst channel were applied, which effectively separated host cells (Fig 2). Further building blocks were added to calculate the intensity of Alexa Fluor 488 within the spots identified by Hoechst, in infected cells. This script was first applied to images to determine the mean fluorescence intensity in the Alexa 488 channel of Hoechst identified parasites, without the addition of the chemical label. The mean plus three times the standard deviation of this intensity was used to define values above background [27,28], thus the minimum intensity above background required to identify parasite DNA which successfully incorporated the chemical label. An input box was incorporated into the script, in which this minimum intensity above background was entered. Parameters of the developed script, “CHEMICAL LABEL INCORPORATION” are shown in S1 Table.

The CHEMICAL LABEL INCORPORATION SCRIPT (number of objects per well):

1. **Number of *T*. *cruzi* amastigote infected cells** (≥5 parasites per cell, identified with Hoechst).
2. **Number of Hoechst identified *T*. *cruzi* parasites** (in infected cells).
3. **Number of parasites incorporating chemical label** (*T*. *cruzi* parasites with Alexa 488 intensity above background, in infected cells).
4. **% chemical label incorporation**. Alexa 488 spots in infected cells (above background) NO / Hoechst spots in infected cells NO *100 = Percentage colocalisation; where NO = number of objects.
5. **Number of host cells**.

**Fig 3:**
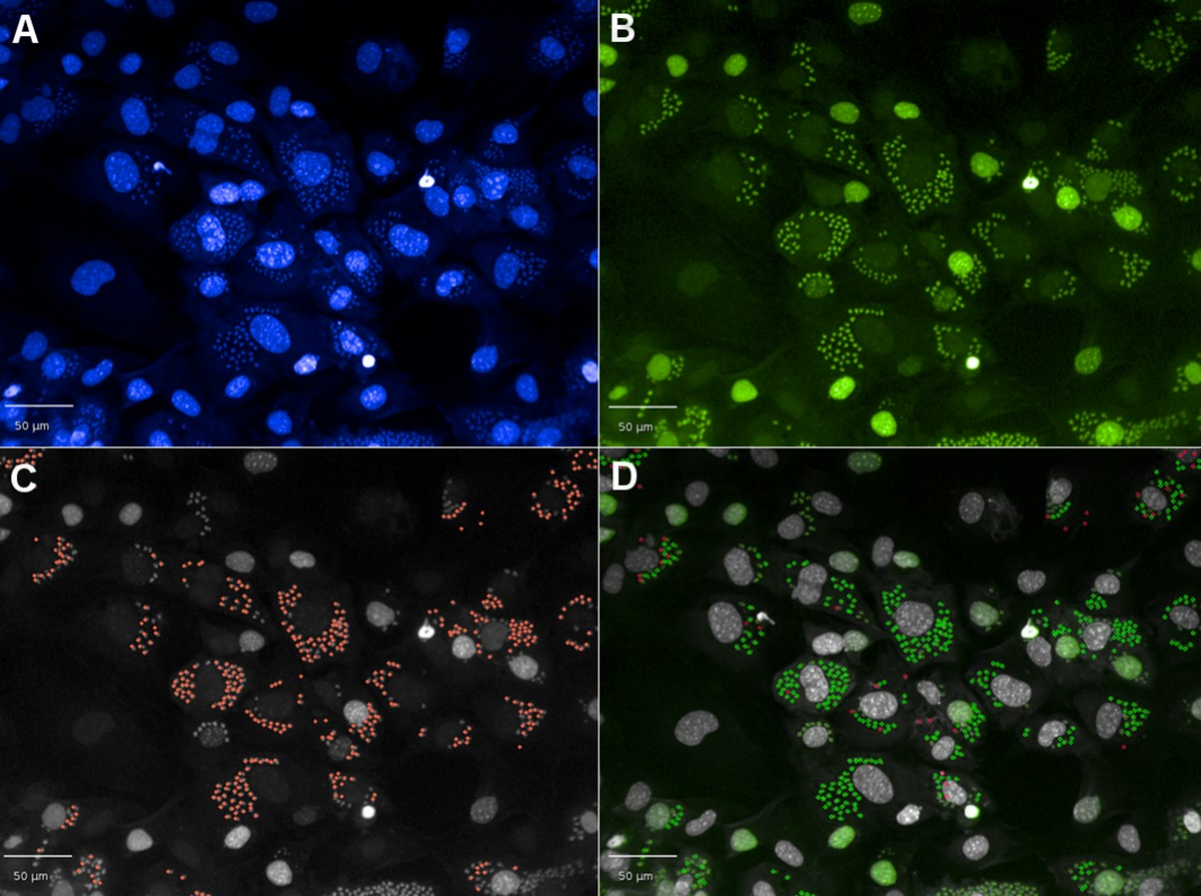
Development of the script to assess the colocalisation of Hoechst and Alexa Fluor 488, using 0.037 μM of EdU. A) Parasite and host cell DNA stained with Hoechst. B) Identification of replicating DNA by exposing infected cells to 0.037 μM of EdU for 48 hours. Images taken in the Alexa 488 channel at 20x magnification. C) Application of the spot detection script to identify parasites in the Hoechst channel that exhibit fluorescence intensity in the Alexa 488 channel (orange spots). D) Parasites from (C) that exhibit Alexa 488 fluorescence above the background (incorporate EdU; green spots), red are parasites that do not exhibit Alexa 488 fluorescence above the background (do not incorporate EdU; red spots).

The script applied to images to identify chemical label incorporation is illustrated in Fig 3. It was determined that 93.1 ± 2.8% of cells contained Alexa Fluor 488 labelled DNA following 48 hours exposure to 0.037 μM of EdU. Below this concentration the percentage of cells with incorporation decreased, equivalent to 72 ± 1.09%, at 0.018 μM of EdU. Above this concentration, the highest incorporation was 94 ± 2.5% at 0.073 μM.

EdU at both 0.037 μM and 0.018 μM resulted in growth inhibition of *T*. *cruzi* amastigotes after 48 hours, with 51 ± 13% and 63 ± 19% inhibition of *T*. *cruzi* growth observed, respectively. Utilising the highest incorporation observed at the lowest concentration of EdC, 89 ± 2.7% of cells had incorporated the DNA chemical label following exposure to 0.73 μM for 48 hours. There was, however, a similar issue with inhibition of *T*. *cruzi* amastigotes exhibited by EdC at concentrations demonstrating DNA incorporation of the analogue, especially when considering E_max_ concentrations that would be required to ensure maximum incorporation. F-ara-EdU was found in 91 ± 0.23% of parasites at the top concentration used (73 μM), whilst there was no incorporation observed (<20%) for Cl-ara-EdU, Br-ara-EdU, and I-ara-EdU with concentrations up to a maximum of 73 μM. To reduce potential toxicity, and to determine if incorporation of EdU, EdC and F-ara-EdU was modified with time, further investigation following shorter periods of 2 and 4 hour incubations was undertaken.

**Fig 4:**
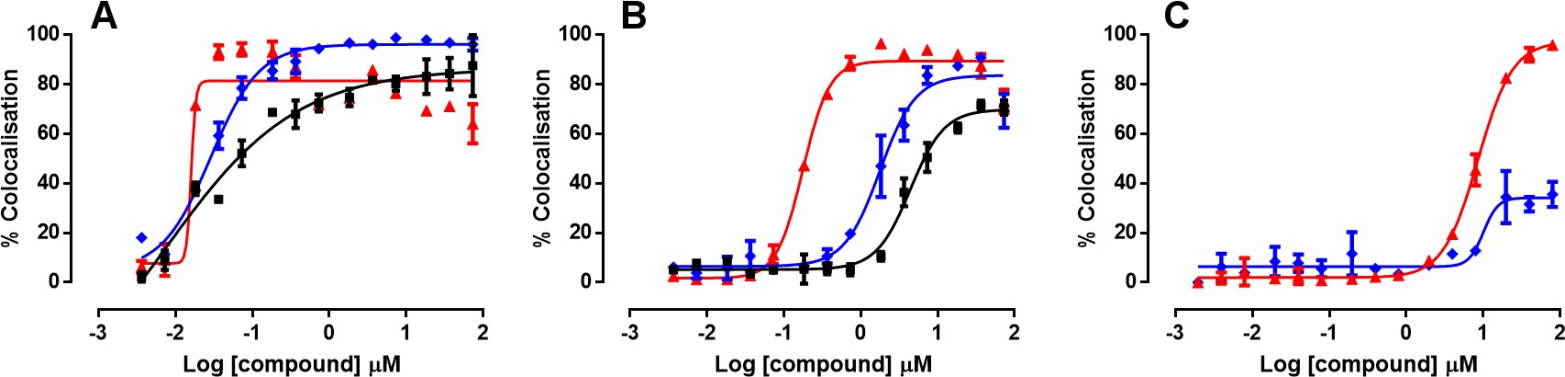
Incorporation of EdU and related analogues EdC and F-ara-EdU into *T*. *cruzi* parasites in infected 3T3 cells over time. Infected cells were exposed to compounds at final assay concentrations ranging from 73 to 8.3 x 10^−4^ μM. A) EdU. B) EdC. C) F-ara-EdU. Red = 48 hours; blue = 4 hours; black = 2 hours exposure to pyrimidine nucleoside analogue.

The percentage colocalisation of EdU and EdC following 48, 4 and 2 hour incubations and F-ara-EdU following 48 and 4 hours are shown in Fig 4. No incorporation of F-ara-EdU into parasite DNA was observed following 2 hours exposure.

E_max_ concentrations were selected to describe the lowest concentration on the plateau of activity where the chemical probes were incorporated, to reduce any potential toxicity of probes on the parasite. Following 4 hours, EdU was detected in 89 ± 4.8% μM of Hoechst defined *T*. *cruzi* cells at 0.37 μM. After 2 hours, EdU showed 75 ± 3.6% incorporation into *T*. *cruzi* DNA at 1.8 μM. 84 ± 3.5% of parasites incorporated EdC within their DNA following 4 hours incubations at 7.3 μM, and after 2 hours incubation, EdC was detected in 71 ± 2.9% parasites with a concentration of 37 μM. After 4 hours incubation, only 36 ± 7.3% of *T*. *cruzi* parasites were observed to incorporate F-ara-EdU at a concentration of 73 μM. Consequently, this analogue was not used in follow up studies.

### EdU as a chemical probe to detect parasite replication following exposure to inhibitors

EdU was used as a chemical probe to determine the effect of compounds, with known actvity against *T*. *cruzi*, on the replication of *T*. *cruzi* parasites, following exposure to compounds for either 2 or 48 hours. The CHEMICAL LABEL INCORPORATION SCRIPT was utilized to assess, within infected cells (defined as ≥5 parasites in a host cell, identified with Hoechst), the number of parasites incorporating chemical label, compared to the number of parasites identified with Hoechst. Following script outputs of Hoechst identified and Alexa 488 incorporating cells, normalized data (to controls) were imported into GraphPad Prism 5, to compare the inhibition of replicating and non- replicating cells, following exposure of *T*. *cruzi* infected cells to known inhibitory compounds. To validate the newly developed method for identification of the number of infected cells, results were compared to a well-established assay ([7]) to determine whether exposure to EdU or the azide click-chemistry cocktail may affect results. The two assays performed with similar sensitivity, with the activity of compounds found to have similar IC_50_ values (Table 2), when compared to our most recent publication [9] there was <2 fold difference in the IC_50_ value of the compounds found.

**Table 2:** The activity of compounds, including two clinically used drugs, against *T*. *cruzi* infection and replication. 1) Infection of 3T3 fibroblasts *in vitro* was determined with a well-established intracellular infection assay [7]. These results were compared to the replication of *T*. *cruzi* measured in 3T3 cells utilising a combination of Hoechst and EdU. For (2), the maximum activity demonstrated with POSA (E_max_) against *T*. *cruzi* was 75%, therefore the IC_50_ value was based on a sub-optimal effect. 3) Significance p<0.05. NR = % of remaining cells that are non-replicating (% amastigotes identified with Hoechst-% amastigotes identified with EdU). R = residual cells that are replicating (in relation to controls). Residual infected cells and residual parasites are calculated from the E_max_ values. NR = not replicating. Cmpd = compound. CF = clemastine fumarate, POSA = posaconazole, NFX = nifurtimox, BZ = benznidazole.

Cmpd	IC_50_ (μM) mean ± SD			Residual parasites at E_max_		
	IC_50_ value (μM)			Residual infected cells	Residual parasites	
	Infected cells (Hoechst)^1^	Amastigotes (Hoechst)	Amastigotes (EdU)	ALL	(Hoechst-EdU) NR	R
**CF**	0.56 ± 0.046	0.43 ± 0.031	0.46 ± 0.10	10	3.5	1.5
**POSA**	0.0040 ± 0.0082^2^	0.0038 ± 0.0010	0.0038 ± 0.0010	25	5.5^3^	2.0
**NFX**	0.80 ± 0.090	0.32 ± 0.024	0.21 ± 0.0018	0	0	0
**BZ**	6.7 ± 0.64	2.80 ± 0.94	1.29 ± 0.25	0	0.50	0

The IC_50_ value determined with the number of Hoechst identified parasites following compound treatment was compared to the IC_50_ value determined with the number of EdU incorporating parasites (Table 2). The E_max_, or maximum activity achieved for each compound [9] was calculated for all parasites (Hoechst) and EdU incorporating parasites, to determine the % of residual parasites following compounds treatment that were replicating and non-replicating (Table 2).

**Fig 5:**
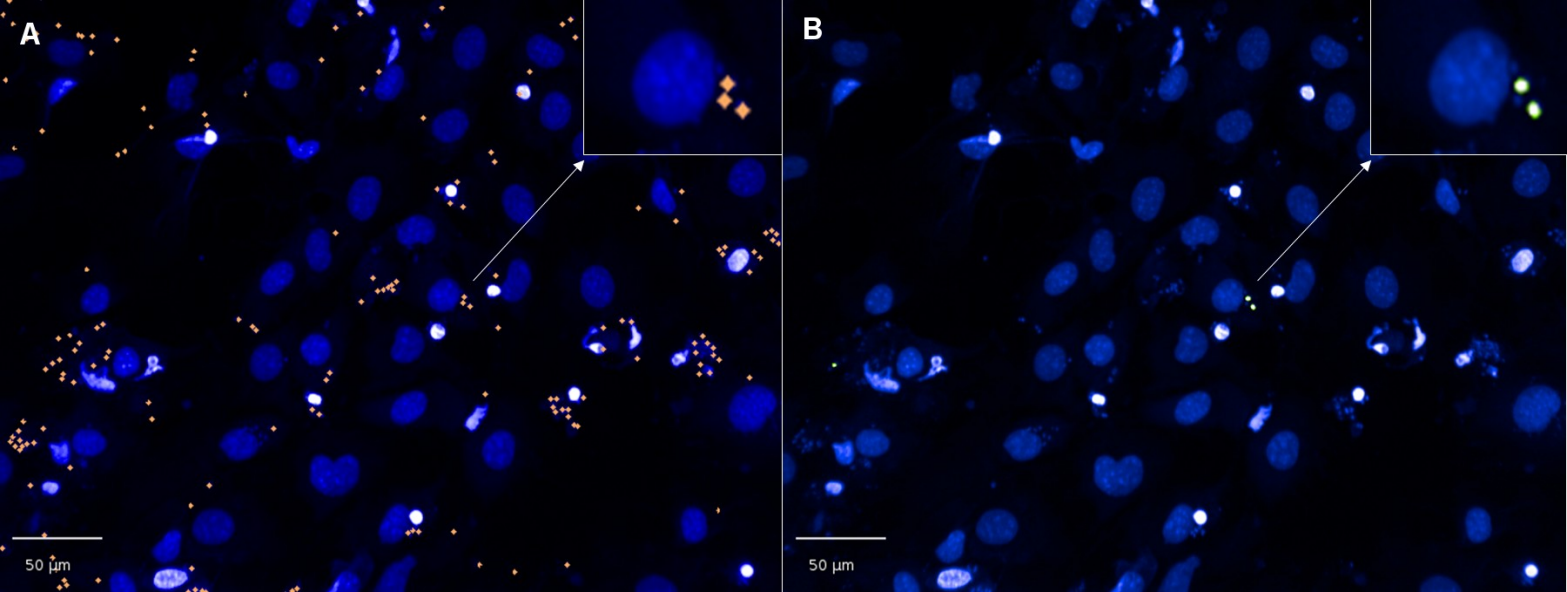
Images of *T*. *cruzi* parasites following exposure to 0.1 μM of POSA for 48 hours. Incorporation was identified with a script developed to enumerate parasites within infected host cells with Hoechst, compared to parasites which incorporated EdU. A small population of replicating parasites was observed in host cells containing <5 parasites. A) Hoechst identified parasites. B) Two parasites, which incorporated EdU (replicating cells, shown in green).

There were 25% of infected cells remaining following POSA treatment. A significant difference in activity (p = 0.0082%) was observed when the E_max_ values obtained for POSA were compared between the number of Hoechst identified parasites and the number of parasites which incorporated EdU, with 5.5% of parasites remaining per well, that were not replicating (Hoeschst identified parasites-EdU identified parasites). At the E_max_, there were 2% of replicating parasites remaining following POSA treatment. However, closer observation of the images indicated there were also some cells that had < 5 parasites (thus not defined as infected) that contained replicating parasites, that had incorporated EdU (Fig 5). Future experiments should therefore increase the magnification and fields of view to more accurately define the level of replicating cells following exposure to POSA. Some differences were also observed in the number of parasites following treatment with CF as we have recently reported [9], with 10% of infected cells not inhibited.

A comparison of IC_50_ values calculated utilising either Hoechst spots or replicating parasites spots (EdU incorporation) revealed no observable differences in the IC_50_ values for CF or POSA, however there was a slight decrease in the IC_50_ value when calculated for EdU versus Hoechst for BZ and NFX, of 1.5 and 2.2 fold, respectively. There was a difference in the activity of BZ and NFX when comparing the IC_50_ value calculated from the number of infected cells to the IC_50_ value calculated from number of Hoecsht identified parasites in infected cells (Table 2). This may be due to a reduction in the number of parasites per cell, that is not captured in determining the number of infected cells. The script used defines 3T3 host cells with ≥5 parasites per cell as infected, thus does not indicate differences in parasite load per cell. The number of parasites per well however defines the total number of amastigotes in infected cells. Compounds may result in less inhibition of the whole number of infected cells, compared to inhibition of the whole number of parasites in infected cells. This could account for the shift in the IC_50_ value for NFX and BZ observed when comparing methods to calculate compound activity. Thus, the numbers of parasites were used for determining both replicating and non-replicating parasites.

Control wells (no inhibition, negative control) were imaged at 60x magnification to identify the typical structure of the parasite following fluorescent staining with Hoechst and/or EdU. This image shows that not all parasites incorporated EdU into their DNA under the experimental conditions used (S1 Fig).

**Fig 6:**
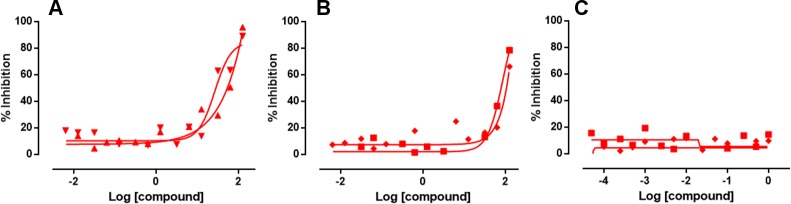
Inhibition of the incorporation of EdU into *T*. *cruzi* DNA by the inhibitors NFX, BZ and POSA, following 2 hours exposure of inhibitor. Inhibitor was removed and parasites were exposed to 1.83 μM of the DNA chemical label, EdU for 2 hours. The incorporation of EdU into parasite DNA was visualised by click chemistry followed by quantification with a script developed in Columbus. A) NFX. B) BZ. C) POSA. Duplicate datasets are represented on the graphs.

Following exposure of *T cruzi* amastigotes to inhibitors for 2 hours at a final assay concentration of 63 μM NFX (80 x the IC_50_ value), 127 μM BZ (20 x the IC_50_ value), the mean inhibition of incorporation of EdU into the parasite DNA was 57 ± 9.1% and 72 ± 8.7%, respectively. In comparison, 1 μM of POSA (250 x the IC_50_ value) resulted in no inhibition of EdU incorporation (Fig 6), in addition to CF resulting in <20% inhibition at 45 x the IC_50_ value. There was no reduction of the number of parasites identified with Hoechst following 2 hours exposure with any of these inhibitors. Data was normalized to positive controls of no addition of EdU and negative controls of 1.83 μM EdU.

## Discussion

The suitability of a panel of six nucleoside analogues as probes to identify replicating intracellular *T*. *cruzi* parasites was assessed. The panel of nucleoside analogues was comprised of the ribosyl thymidine and cytidine analogues EdU and EdC, respectively, and arabinosyl thymidine analogues featuring halogenation (fluoro, chloro or iodo) at the 2′ position. The ribosyl analogues were metabolised and incorporated into parasite DNA, reflecting structural similarity to the native nucleosides thymidine and cytidine. The 2′-halogenated arabinosyl nucleoside analogues were proposed to be less cytotoxic than EdU, as a function of increased aglycone linkage stability resisting base excision DNA repair pathways [29]. These analogues were proposed to also exhibit decreased metabolic activity compared to EdU as the steric bulk at the 2′ position increased from fluoro to chloro to iodo substituents. The incorporation profiles were confirmed experimentally for the six nucleoside chemical probes tested. EdU, EdC and F-ara-EdU incorporated into *T*. *cruzi* DNA following 48 hours incubation, while Cl-ara-EdU, Br-ara-EdU and I-ara-EdU did not.

The inhibitory activity exhibited by EdU against HEK293 cells and *T*. *cruzi* has been similarly shown for a number of cell lines both *in vivo* and *in vitro* [30,31]. For example, it has been demonstrated that following 72 hours exposure of HEK293 cells to EdU there was cytotoxicity displayed, with an IC_50_ value of 0.060 ± 0.043 μM identified [32]. A recent report has described lack of toxicity of EdU toward *L*. *donovani* following 72 hours incubation [20]. It could be that these parasites have different methods of transport / variability in enzymes for nucleoside analogue incorporation into their DNA. Additionally, the THP-1 cells used as the *L*. *donovani* host cell may not be as sensitive to the effects of EdU as replicating cell lines, such as HEK293, as differentiated THP-1 cells have a very low level to no cell division [33]. Similarly, the 3T3 cells utilised in the studies herein are subject to contact inhibition, which could explain why EdU did not show activity against 3T3 cells following 48 hours incubation. This finding highlights the benefit of using proliferating cell lines when assessing compound activity. Considering the effect of EdU and EdC against *T*. *cruzi* / HEK293 cells following 48 hours incubation, the utilisation of pyrimidine nucleoside analogues which do not affect the growth of *T*. *cruzi* holds potential for long exposure chemical probe applications. The 2ʹ-halogenated arabinosyl nucleoside analogues did not inhibit growth of the parasite nor HEK293 cells as predicted, however did not incorporate into parasite DNA.

To reduce or abrogate potential issues of growth inhibition with EdU and EdC toward *T*. *cruzi*, the incubation time with these probes was reduced to 2 and 4 hours, which corresponded to no observed inhibitory activity against *T*. *cruzi* intracellular amastigotes. In these studies, EdC also exhibited toxicity toward *T. cruzi*, perhaps as deamination of EdC monophosphate leads to the production of EdU [34]. Due to the amount of F-ara-EdU needed to show incorporation following 48 hours incubation, this compound was not investigated over a shorter exposure period. The incubation time was reduced for EdU as a DNA probe to detect replication in mammalian cells in a similar effort to reduce the toxic effects of this compound [34]. The percentage level of incorporation of EdU into parasites following 2 hours exposure was increased in comparison to a report utilising cancer cell lines [23]. It may be that in comparison, the S-phase is common in asynchronous *T*. *cruzi* parasites even after a short incubation, or potentially due to transporter / metabolism of EdU. The effects of clinically used drugs and compounds, with known activity against *T*. *cruzi*, were determined on parasite replication, using EdU as a DNA probe. Compounds with known activity against the parasite included the drugs currently used to treat Chagas disease, NFX and BZ, in addition to POSA which was not efficacious in pre-clinical trials [35]. The drugs used to treat Chagas disease do not have a specifically defined target, however are thought to undergo reduction to unstable intermediates that fuel oxidative stress and cause DNA damage [36–38]. CF does not have a known *T*. *cruzi* mode of action or target defined.

From our previous research, we have shown POSA exhibits a sub-efficacious effect upon *T*. *cruzi* parasites identified with Hoechst following 48 hours incubation [7,9]. These parasites are viable *in vitro* following long-term incubation without compound pressure [9]. Using EdU to assess parasite replication, it was found that a statistically significant number of this population were not replicating. It may be that that the majority of cells remaining may be very slowly replicating, are apoptotic, or quiescent. As EdU identifies parasites that are in the S-phase of cell division, these POSA exposed parasites may be in a different phase of the cell cycle. Methods have been published to determine pre- and post S-phase in *L*. *mexicana* asynchronous promastigotes utilising total cellular DNA content and kinetoplast to nuclear counts [39], requiring flow cytometry. A recent study has also analysed the cell cycle of *T*. *cruzi* intracellular amastigotes with DAPI, utilising host cell lysis followed by flow cytometry, with Tulahuen strain parasites expressing β-galactosidase [40]. Tulahuen strain epimastigotes cannot be used as an axenic surrogate for cell cycle studies as they are not sensitive to POSA, even up to 120 hours exposure [9]. However, it should be noted that epimastigotes of the Y strain of *T*. *cruzi* have been reported as sensitive to POSA [41] and thus may serve as an alternative strain. Recent profiling of the *T*. *cruzi* transcriptome during the proliferative cycle has identified a number of cell cycle regulated mRNAs which in future could lead to identification of the molecular networks driving parasite specific proliferation [42] to support DNA chemical probe studies. It could also be that non-replicating cells are cell cycle arrested by CYP51 inhibition related to alterations in the composition of lipids, as has been suggested following POSA treatment in *L*. *amazonensis* [43]. Importantly, a low replicative state could contribute to issues with POSA treatment of Chagas disease.

Recent discussions highlight that in clinical trials with POSA for treatment of Chagas disease, potential exists that the dosing regimen may have resulted in the lack of clinical efficacy of this compound [44]. It is suggested that the efficacy of POSA could be directly related to drug exposure, rather than concentration-dependence and additionally, pharmacokinetic studies conducted in the POSA clinical trials revealed a lower drug exposure compared with the animal models [44]. Whilst the dosing of POSA in clinical trials of 400 mg/kg twice daily for 60 days was not successful [43], POSA could be a partner drug in combination therapy with new or known compounds in the future [44,45], especially since POSA may reduce toxicity and shorten duration of treatment of drugs such as BZ, which although is a fast-acting, has toxic side effects [35]. It is important to classify slow-acting compounds in drug discovery efforts for Chagas disease, so that increased dosing can be investigated to maintain a sustained exposure *in vivo*, coupled with rigorous PK/PD analyses to translate the preclinical results to clinical development [46]. Additionally, treatment combinations may be investigated. The methods utilising EdU as a chemical label to identify replicating *T*. *cruzi* DNA outlined herein support classification of POSA as a slow-acting compound and provide a valuable tool to support classification of compounds with similar MOA. Other potentially slow-acting molecules have recently been identified as pan-active against kinetoplastid species including *T*. *cruzi*, such as proteasome inhibitors [47]. To address a bias for identifying and progressing fast-acting compounds using the method described herein, future studies will investigate longer exposure of *T*. *cruzi* to compounds (>72 hours), in addition to the relatively short period of 48 hours used here, to provide a more balanced and comprehensive study design.

To identify the effect of the nucleoside analogues on the survival of parasites, important for understanding the limits of incubation, the activity for different incubation times was determined. Whilst EdU and EdC displayed activity following 24 hours incubation, the inhibition did not reach 100% following either 24 or 48 hours incubation. Following compound wash off, there was still incomplete clearance of parasites, however there was little change in the % of parasites remaining at E_max_ concentrations for EdU. There was however, a shift in the IC_50_ value, with marked reduction in activity, illustrating that at lower concentrations, parasites grow out. Following wash off, parasites exposed to EdC were able to grow out at all concentrations and an IC_50_ value could not be determined. This does not necessarily describe a static MOA for these compounds. EdU does not incorporate into all parasites, shown by a maximum of 94% incorporation following 48 hours incubation. It could be possible that some slow growing parasites may be refractive to exposure to EdU following 48 hours exposure, as seen with POSA. Whilst EdU and EdC incorporation is fast, it is inhibitory to *T*. *cruzi* Tulahuen strain parasites and supports the limited exposure required for EdU in further studies.

Following exposure of parasites to CF there were 10% of infected host cells present at E_max_ concentrations. Similarly, we have previously shown 3% of *T*. *cruzi* infected cells, identified with Hoechst, remaining following CF treatment for 48 hours [7]. Very few parasites remaining following compound exposure were shown to be replicating at E_max_ concentrations. Further studies at higher magnification, as suggested for POSA, are required to quantify replicating parasite levels. Replicating parasites identified remaining following CF treatment may contribute to the previously reported failure of this compound in a *T*. *cruzi* mouse model of infection [48], however further studies are needed to confirm this.

We investigated 2 hours incubation with NFX, BZ and POSA as it would be expected lower exposure would cause less cell death and may be useful to tease out cell potential cycle inhibition / DNA damage by this class of compound. Whilst it can’t currently be concluded, DNA damage / cell cycle inhibition by NFX and BZ may be supported by a reduction in incorporation of EdU following 2 hours exposure, and the previously reported activity of these compounds on DNA [49]. Additionally, this shows a fast action by these compounds. There was no effect on EdU incorporation by POSA following 2 hours exposure, likely due to the slow-acting nature of POSA [9]. We have previously shown NFX and BZ to be fast-acting in comparison to *T*. *cruzi* CYP51 inhibitors [7]. Whilst it has been suggested that POSA is slow-acting by *in vivo* studies [50] and *in vitro* studies [11], this is the first time that NFX has shown an effect on *T*. *cruzi* replication following a very short incubation *in vitro*. Rapid inhibition of replication could be an important hallmark of an effective compound *in vivo*. Determining compound effects against *T*. *cruzi* replication enables the clear identification of fast and slow-acting compounds for classification in the drug discovery pipeline. This can be extrapolated to longer incubations to determine if very small populations of parasites are able to survive following compound exposure, which could result in lack of efficacy *in vivo*.

Nucleoside incorporation into *T*. *cruzi* DNA lends itself to future investigations, with less toxic compounds. Recently, Sánchez-Valdéz and colleagues (2018) utilised EdU as a DNA chemical label to identify *T*. *cruzi* DNA replication suggesting there may be dormant cells during *T*. *cruzi* infection both *in vitro* and *ex vivo* [51]. However, these cells may be more slowly replicating and thus in potentially different cell cycle phases, and the time of exposure could exhibit growth inhibition against the parasite. In their study, it was determined with division tracker dyes that EdU did not inhibit parasite replication following 72 hours, although there were not controls without EdU to illustrate this. However, a different parasite strain and host cells in the study herein (Tulahuen strain parasites) compared to the 2018 study (Colombiana, Brazil, CL3 and ARC0704 strains) could impact the effects of EdU on the parasites. Some strains used by Sánchez-Valdéz et al were transfected with pTREX-Luciferase/ TdTomato and it would be of benefit to compare with wild type parasites. The impact of EdU causing time dependant inhibition of cell growth on various mammalian cell lines is well documented [52,53]. In the studies herein, exposure of EdU was limited to 2 or 4 hours incubation. Whilst recent studies also utilise 2 hours exposure of EdU to determine replicating *T*. *cruzi* parasites [19], longer exposures would also be warranted to assess the impact on parasite number. These studies show lack of efficacy of 1 μM POSA against replicating *T*. *cruzi* observed against Silvio and PAH179 intracellular amastigotes, following 5 days incubation [19], and POSA did not appear to reach E_max_ at the doses tested against PAH179 parasites, utilising Hoechst to identify parasite numbers. These recent studies demonstrate that EdU can be successfully utilised across strains and herein we show that both Hoechst and EdU can be used to assess multiple compound doses to identify the activity of compounds against *T*. *cruzi* replication.

This current study is important to highlight the effect of EdU on the growth of *T*. *cruzi* for future research in replication studies. Investigations into additional more selective nucleoside analogues are warranted as *T*. *cruzi* DNA chemical probes which facilitate long exposure times. It would be advantageous to understand in more detail the replication of *T*. *cruzi* parasites during the chronic stage of the disease, with probes which may exhibit less parasite growth inhibition. Next generation chemical probes with improved properties will increase the potential for studying DNA synthesis and reduction of the inhibition of thymidylate synthase, mediated by EdU [53] and could reduce the effects of this compound on parasite growth.

## Supporting information

S1 Table Flow and steps in the CHEMICAL LABEL INCORPORATION SCRIPT. The script is first run on *T*. *cruzi* infected cells with addition of Hoechst and no addition of EdU, to identify the background intensity of parasites, in the Alexa 488 channel (488 nm laser; 540/75 nm emission). The host cell cytoplasm is identified in the far red channel (640 nm laser; 690/50 emission). The script is then run again, and the background Alexa 488 background is added as an input (> intensity). The number of parasites identified in the Hoechst channel (405 nm laser; 450/50 nm emission) and incorporating the chemical label is calculated (> background). See output for the data generated. All measurements are the total number per well. The nuclei detection algorithm, cytoplasm and spot detection are C, A and C, respectively, are specific to Columbus (PerkinElmer).
(DOCX)

S1 Fig Images of *T*. *cruzi* amastigotes in host 3T3 cells stained with the nuclear marker Hoeschst (blue) and replicating cells identified with EdU (green). The host cell cytoplasm is identified with CellMask Deep Red Plasma Membrane stain. Images captured at 60x magnification. A) Hoechst (blue). B) EdU (green). K = kinetoplast, N = nucleus. C) Hoechst and EdU multiplexed with CellMask Deep Red Plasma Membrane stain.
(DOCX)
