# Supplementary material for: Investigation of pyrimidine nucleoside analogues as chemical probes to assess compound effects on the proliferation of Trypanosoma cruzi intracellular parasites
Source: PLoS Negl Trop Dis. 2020 Mar 12;14(3):e0008068. doi: 10.1371/journal.pntd.0008068 (PMC7112222; doi:10.1371/journal.pntd.0008068)
Supplement: S1 Table — The script is first run on T. cruzi infected cells with addition of Hoechst and no addition of EdU, to identify the background intensity of parasites, in the Alexa 488 channel (488 nm laser; 540/75 nm emission). The host cell cytoplasm is identified in the far red channel (640 nm laser; 690/50 emission). The script is then run again, and the background Alexa 488 background is added as an input (> intensity). The number of parasites identified in the Hoechst channel (405 nm laser; 450/50 nm emission) and incorporating the chemical label is calculated (> background). See output for the data generated. All measurements are the total number per well. The nuclei detection algorithm, cytoplasm and spot detection are C, A and C, respectively, are specific to Columbus (PerkinElmer). (DOCX) [file pntd.0008068.s001.docx]

|  | CHEMICAL LABEL INCORPORATION SCRIPT | | |
| --- | --- | --- | --- |
|  | **CELL COMPONENT** |  | **VARIABLE** |
| Find Nuclei (channel=Hoechst) | |  |  |
|  | Nuclei Detection Algorithm: |  | C |
|  | Common Threshold: |  | 0.1 |
|  | Nuclear Area (µm²): |  | >100 |
|  | Population |  | ALL NUCLEI |
|  | Calculate morphology properties |  |  |
|  | In population |  | All nuclei |
|  | Nucleus area (µm²) |  | >30 |
|  | Nucleus roundness |  | >0.6 |
|  | Population |  | NUCLEI SELECTED |
| Find Cytoplasm (channel=Far Red) | |  |  |
|  | In population |  | Nuclei selected |
|  | Method: |  | A |
|  | Select cell region (no nucleus) |  |  |
|  | OuterBorder: |  | 0% |
|  | InnerBorder: |  | 50% |
|  | Population |  | HOST CELLS |
| Find Spots (channel=Hoechst) | |  |  |
|  | In population |  | Host cells |
|  | Region: |  | Cytoplasm |
|  | Method: |  | C |
|  | Spot Radius: |  | ≤2.2 |
|  | UnitType: |  | px |
|  | Contrast: |  | <0.22 |
|  | Uncorrected spot to region intensity: |  | >2.2 |
|  | Number of spots (select population) |  | ≥5 |
|  | Output |  | INFECTED CELLS |
|  | Output |  | SPOTS IN INFECTED CELLS |
| Calculate intensity properties (channel=Alexa 488) | |  |  |
|  | In population |  | Spots in infected cells |
|  | Output (intensity spot mean) |  | ALEXA 488 SIGNAL |
| Find spots incorporating EdU (channel=Alexa 488) | |  |  |
|  | In population |  | Spots in infected cells |
|  | **Input background Alexa 488 signal**  **(select population)** |  | > |
|  | Output |  | SPOTS INCORPORATING EDU |
|  | Output |  | % SPOTS INCORPORATING EDU |

**Table S1.** Flow and steps in the CHEMICAL LABEL INCORPORATION SCRIPT. The script is first run on *T. cruzi* infected cells with addition of Hoechst and no addition of EdU, to identify the background intensity of parasites, in the Alexa 488 channel (488 nm laser; 540/75 nm emission). The host cell cytoplasm is identified in the far red channel (640 nm laser; 690/50 emission). The script is then run again, and the background Alexa 488 background is added as an input (> intensity). The number of parasites identified in the Hoechst channel (405 nm laser; 450/50 nm emission) and incorporating the chemical label is calculated (> background). See output for the data generated. All measurements are the total number per well. The nuclei detection algorithm, cytoplasm and spot detection are C, A and C, respectively, are specific to Columbus (PerkinElmer).
