# Supplementary material for: Investigation of pyrimidine nucleoside analogues as chemical probes to assess compound effects on the proliferation of Trypanosoma cruzi intracellular parasites
Source: PLoS Negl Trop Dis. 2020 Mar 12;14(3):e0008068. doi: 10.1371/journal.pntd.0008068 (PMC7112222; doi:10.1371/journal.pntd.0008068)
Supplement: S1 Fig — Images of T. cruzi amastigotes in host 3T3 cells stained with the nuclear marker Hoeschst (blue) and replicating cells identified with EdU (green). The host cell cytoplasm is identified with CellMask Deep Red Plasma Membrane stain. Images captured at 60x magnification. A) Hoechst (blue). B) EdU (green). K = kinetoplast, N = nucleus. C) Hoechst and EdU multiplexed with CellMask Deep Red Plasma Membrane stain. (DOCX) [file pntd.0008068.s002.docx]

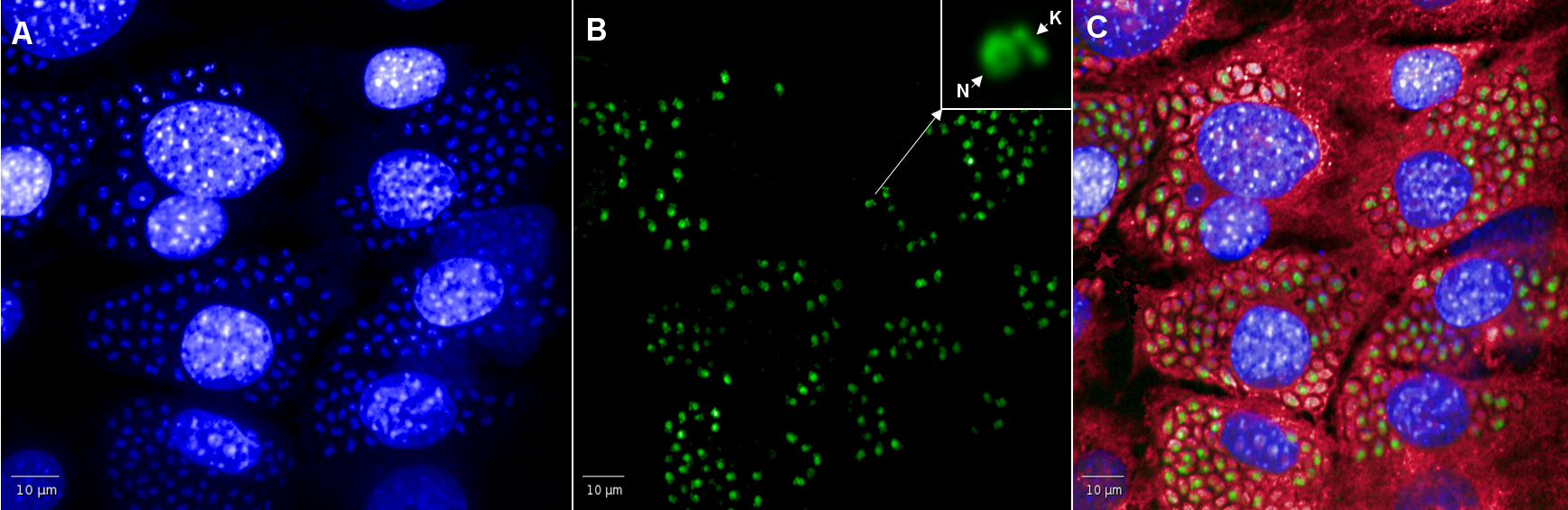


**Figure S1****.** Images of *T. cruzi* amastigotes in host 3T3 cells stained with the nuclear marker Hoeschst (blue) and replicating cells identified with EdU (green). The host cell cytoplasm is identified with CellMask Deep Red Plasma Membrane stain. Images captured at 60x magnification. A) Hoechst (blue). B) EdU (green). K=kinetoplast, N=nucleus. C) Hoechst and EdU multiplexed with CellMask Deep Red Plasma Membrane stain.
